# Panobinostat-loaded folate targeted liposomes as a promising drug delivery system for treatment of canine B-cell lymphoma

**DOI:** 10.3389/fvets.2023.1236136

**Published:** 2023-08-30

**Authors:** Ana S. André, Joana N. R. Dias, Sandra I. Aguiar, Ana Leonardo, Sara Nogueira, Joana D. Amaral, Célia Fernandes, Lurdes Gano, João D. G. Correia, Marco Cavaco, Vera Neves, Jorge Correia, Miguel Castanho, Cecília M. P. Rodrigues, Maria Manuela Gaspar, Luís Tavares, Frederico Aires-da-Silva

**Affiliations:** ^1^Faculty of Veterinary Medicine, CIISA-Centre for Interdisciplinary Research in Animal Health, University of Lisbon, Avenida da Universidade Técnica, Lisbon, Portugal; ^2^Associate Laboratory for Animal and Veterinary Sciences (AL4AnimalS), Lisbon, Portugal; ^3^Research Institute for Medicines (iMed.ULisboa), Faculty of Pharmacy, Universidade de Lisboa, Lisbon, Portugal; ^4^Departamento de Engenharia e Ciências Nucleares, Centro de Ciências e Tecnologias Nucleares, Instituto Superior Técnico, Universidade de Lisboa, CTN, Bobadela, Portugal; ^5^Faculdade de Medicina, Instituto de Medicina Molecular-João Lobo Antunes, Universidade de Lisboa, Lisbon, Portugal

**Keywords:** non-Hodgkin lymphoma, canine B-cell lymphoma, liposome, folate, panobinostat, drug delivery

## Abstract

**Introduction:**

Cancer is a major public health problem with over 19 million cases reported in 2020. Similarly to humans, dogs are also largely affected by cancer, with non-Hodgkin's lymphoma (NHL) among the most common cancers in both species. Comparative medicine has the potential to accelerate the development of new therapeutic options in oncology by leveraging commonalities between diseases affecting both humans and animals. Within this context, in the present study, we investigated the potential of panobinostat (Pan)-loaded folate-targeted PEGylated liposomes (FA-PEG-Pan-Lip) for the treatment of canine B-cell lymphoma, while contributing to new perspectives in comparative oncology.

**Methods and results:**

Two formulations were developed, namely: PEG-Pan-Lip and FA-PEG-Pan-Lip. Firstly, folate receptor expression in the CLBL-1 canine B-cell lymphoma cell line was assessed. After confirming receptor expression, both Pan-loaded formulations (PEG-Pan-Lip, FA-PEG-Pan-Lip) demonstrated dose-dependent inhibitory effects on CLBL-1 cell proliferation. The FA-PEG-Pan-Lip formulation (IC_50_ = 10.9 ± 0.03 nM) showed higher cytotoxicity than the non-targeted PEG-Pan-Lip formulation (IC_50_ = 12.9 ± 0.03 nM) and the free panobinostat (Pan) compound (IC_50_ = 18.32±0.03 nM). Moreover, mechanistically, both Pan-containing formulations induced acetylation of H3 histone and apoptosis. Flow cytometry and immunofluorescence analysis of intracellular uptake of rhodamine-labeled liposome formulations in CLBL-1 cells confirmed cellular internalization of PEG-Lip and FA-PEG-Lip formulations and higher uptake profile for the latter. Biodistribution studies of both radiolabeled formulations in CD1 and SCID mice revealed a rapid clearance from the major organs and a 1.6-fold enhancement of tumor uptake at 24 h for ^111^In-FA-PEG-Pan-Lip (2.2 ± 0.1 %ID/g of tumor) compared to ^111^In-PEG-Pan-Lip formulation (1.2±0.2 %ID/g of tumor).

**Discussion:**

In summary, our results provide new data validating Pan-loaded folate liposomes as a promising targeted drug delivery system for the treatment of canine B-cell lymphoma and open innovative perspectives for comparative oncology.

## Introduction

Cancer is a major public health and economic issue, and its burden continues to increase worldwide. With over 19 million cases in 2020, it is expected that there will be 29 million cases by 2040 due to aging and growing population (1). The past few decades have seen unprecedented advances in the development of new cancer treatments, particularly with the major advances in immunotherapy and the approval of emerging therapeutics, such as immune-checkpoint inhibitors, antibody-drug conjugates, bispecific antibodies, and CAR-T cells. Nevertheless, although the landscape of cancer treatment has changed dramatically in recent years, new approaches to fight cancer need to be explored rapidly and effectively. Comparative medicine has the potential to accelerate the development of new therapeutic options in the field of oncology by leveraging commonalities between diseases that are common to humans and animals. In particular, the canine model provides a powerful resource for developing models of naturally occurring tumors, that share many clinical and pathophysiological features with their human counterparts (2, 3). Domestic dogs are highly affected by cancer and approximately 4 million dogs die from cancer each year, making it the leading cause of death (2). Thus, efforts are also being made in comparative research to provide quality cancer treatment options for dogs as caregivers are becoming increasingly demanding.

Non-Hodgkin's lymphoma (NHL) is one of the most common cancers in both species (2–5). NHL is a malignancy that originates from cells of the immune system, the vast majority of which are B lymphocytes (5). In humans, NHL is among the 15 most prevalent and deadly malignancies worldwide (1). The incidence in dogs is similar to that in humans, affecting 15–30 per 100 000 dogs (2). Owing to the great similarity in pathologic presentation shared between canine and human NHL, the World Health Organization (WHO) classification criteria is also used for canine tumors (3, 6). Although NHL encompasses several subtypes, diffuse large B-cell lymphoma (DLBCL) accounts for one third of all NHL, making it the most common aggressive form in both humans and dogs (2, 6, 7). Veterinary therapies have evolved with human therapies, and similar to humans, CHOP (cyclophosphamide, doxorubicin, vincristine, and prednisolone)-based chemotherapy is the standard treatment for canine lymphoma. Although treatment response and resistance also present (8–11) clinical patterns comparable to human NHL, the low-dose chemotherapeutic protocol used in dogs significantly reduces cure rates in veterinary medicine. In most cases disease relapse occurs after remission and the 2-year survival rate is only 20%, demonstrating the urgent need for novel treatment strategies (5, 7, 12). Moreover, the toxicity of conventional chemotherapy often limits its efficacy. Therefore, interest in designing and developing more targeted and specific molecules has increased over recent years.

Cancer is highly associated with genetic alterations, with epigenetic processes playing a key role in carcinogenesis, namely influencing gene transcription, regulating anti-oncogenes and DNA repair genes. Therefore, new research and discoveries have been directed toward the development of agents that can regulate these epigenetic mechanisms. Among the compounds targeting epigenetic regulators, histone deacetylase inhibitors (HDACis) have emerged as a promising new class of anticancer therapeutics (13). Histone deacetylases are important naturally occurring enzymes that promote deacetylation of histones and alter gene transcription. HDACis act on a variety of proteins mainly involved in the control of cell growth, differentiation, and apoptosis. On the other hand, by inducing acetylation of histone and non-histone proteins, HDACis promote cell differentiation, cell cycle arrest, angiogenesis inhibition and apoptosis induction (13–15). The activity of HDACis has been demonstrated in a number of hematological malignancies, including lymphoblastic leukemia, cutaneous T-cell lymphoma, DLBCL, Hodgkin lymphoma and Burkitt lymphoma (16). Currently, three HDACis are approved by the U.S. Food and Drug Administration (FDA) for clinical use in human cancer therapy: vorinostat, romidepsin, and belinostat. Only belinostat and panobinostat (Pan) have been approved by the European Medicines Agency (EMA) (17–20). Considering the high efficacy of HDACis in human targeted cancer therapy, we recently conducted the first investigation on their antitumor properties using a canine B-cell lymphoma model. For this purpose, a panel of seven HDACis (CI-994, Pan, SBHA, SAHA, scriptaid, trichostatin A and tubacin) were initially tested on the well-characterized CLBL-1 canine B-cell lymphoma cell line, and Pan was identified as the most promising compound with strong *in vitro* and *in vivo* antitumor properties (21). Our results have validated HDACis, and in particular, Pan as a novel anticancer therapy for veterinary medicine, while contributing to comparative oncology. Nevertheless, owing to their potent and broad-spectrum inhibition, HDACis have been associated with significant dose-limiting toxicities, which might lead to some limitations, clinical utility, and safety as a single/adjuvant agent. There are many ways to mitigate the toxicity presented by HDACi, such as the synthesis of more efficient and safer molecules, modification of existing molecules and exploration of drug delivery systems to specifically deliver the HADCi into cancer cells, such as liposomes.

Nanomedicine and drug delivery systems play a prominent role in modern medicine and can help to circumvent the current pitfalls of several anticancer drugs, including non-targeted HDACis. Lipid-based nanosystems, particularly liposomes, represent an attractive nanocarrier for drug delivery for cancer treatment (22–25). Liposomes are lipid vesicles composed of one or more bilayers enclosing one or various internal aqueous compartments that are able to incorporate both hydrophilic and hydrophobic compounds. Liposomes have many advantages such as biodegradability, biocompatibility, improvement of pharmacokinetic profiles, low cytotoxicity and the ability to be modified to allow pH and temperature sensitive release (23, 26, 27). Moreover, due to their unique properties, liposomes can be designed to deliver active drugs to specific sites, through surface modification. In recent years, several ligands, such as monoclonal antibodies, antibody fragments, proteins, peptides, vitamins, carbohydrates, and glycoproteins, have been attached to the surface of liposomes to selectively target tumor cells overexpressing a specific cell surface receptor (8–11). The folate receptor (FR) has been identified as a promising target because it is highly overexpressed on the surface of a variety of tumor types, while its distribution in normal tissues and organs is limited. Some studies have shown that conjugation of folic acid (FA) is a promising approach for active targeting of liposomes to increase the amount of drug delivered to the target cell compared to free drugs or passively targeted liposomes (9, 10, 28). Within this context, in the present study we aimed to develop Pan-loaded folate targeted PEGylated liposomes with improved therapeutic outcomes for the treatment of canine B-cell lymphoma. For this purpose, non-targeted Pan-loaded and folate-targeted PEGylated liposomal formulations were prepared and their cytotoxic and targeting properties were thoroughly investigated.

## Materials and methods

### Materials

Dipalmitoyl phosphatidyl choline (DPPC), poly(ethylene glycol) (PEG-2000) covalently linked to distearoyl phosphatidyl ethanolamine (DSPE-PEG), rhodamine covalently linked to phosphatidyl ethanolamine (Rho-PE) and the functionalized DSPE-PEG phospholipids with folate (DSPE-PEG-FA) were purchased from Avanti Polar Lipids (Alabaster, AL, USA). Cholesterol (Chol), and phosphate buffered saline (PBS) were obtained from Sigma-Aldrich (St. Louis, MO, USA). Pan was purchased from Selleckchem (Houston, TX USA, Cat # S1030). All other reagents were of analytical grade.

### Liposomes preparation

Encapsulation of Pan in liposomes was achieved by an active loading method with an ammonium sulfate gradient as previously described by us (10). Briefly, the relevant lipids, DPPC: Chol: DSPE-PEG in a molar ratio of 1.85: 1: 0.15 for non-targeted liposomes and DPPC: Chol: DSPE-PEG: DSPE-PEG-FA in a molar ratio of 1.85: 1: 0.12: 0.03 for targeted liposomes were dissolved in chloroform and the organic solvent was removed by rotary evaporation. The homogeneous lipid film formed was hydrated with water and the resulting suspension was frozen (-70°C) and lyophilized (Edwards, CO, USA) overnight. Rehydration of the lyophilized powder was performed with ammonium sulfate (135 mM, pH 5.4) at 45°C for 30 min. To produce a homogeneous liposome suspension, the unloaded liposomes were filtered under nitrogen pressure (10–500 lb/in2), through polycarbonate membranes of proper pore size (at 45°C), using a Lipex thermo-barrel extruder (Lipex: Biomembranes Inc., Vancouver, BC, Canada) until the liposomes reached a mean size of 0.1 μm. An ammonium sulfate gradient was established by replacing the extraliposomal medium with PBS buffer (pH 7.4) using a desalting column (Econo-Pac 10 DG, Bio-Rad, Hercules, CA, USA). Pan was incubated with unloaded liposomes at a molar ratio of 1:16 μmol of lipid, previously diluted in PBS (from a stock solution at 67 mg/mL) for 1 h at 45°C. To separate the unencapsulated Pan an ultracentrifugation was performed at 250,000 g for 2 h at 15°C in a Beckman LM-80 ultracentrifuge (Beckman Instruments, Fullerton, CA, USA). The pellet was suspended in PBS (pH 7.4). Four different formulations were prepared: folate-targeted unloaded liposomes (FA-PEG-Lip), non-targeted unloaded liposomes (PEG-Lip), folate-targeted loaded with Pan liposomes (FA-PEG-Pan-Lip) and non-targeted loaded with Pan liposomes (PEG-Pan-Lip).

For flow cytometry studies unloaded liposomes and Pan liposomes were prepared as above described. The only difference was the inclusion in the lipid composition of Rho-PE at 0.2 mol% of total lipid.

For biodistribution studies, selected Pan liposomes were labeled with Indium-111 (^111^In). For that, the chelating agent diethylenetriamine pentaacetic acid (DTPA) at a concentration of 6 μM was encapsulated during liposome preparation after achievement of the lipid film and before lyophilization (29). Then liposomes were prepared as above described. Pan liposomes co-loaded with DTPA were then labeled with ^111^In using the lipophilic complex ^111^In-oxine as precursor, as describe below.

### Characterization of panobinostat liposomal formulations

After disruption of liposomes with ethanol, Pan was quantified by spectrophotometry with the aid of a calibration curve (standards ranged from 2.5 to 20 μg/mL). The absorbance of all samples were read at 282 nm. The lipid content of liposomal formulation under study was determined using an enzyme-linked colorimetric method, Phospholipids Choline Oxidase-Peroxidase (Spinreact, Spain) (30). Liposomes were characterized in terms of lipid composition and by the following encapsulation parameters: the initial and final Pan to lipid ratios [(Pan/Lip)i and (Pan/lip)f, respectively]; and encapsulation efficiency defined as the percentage of [(Pan/Lip)f]/[(Pan/Lip)i]. Pan liposomes mean size was determined by dynamic light scattering in a Zetasizer Nano S (Malvern Instruments Inc., Malvern, UK). As a measure of particle size distribution of the dispersion, the system reports the polydispersity index ranging from 0.0 for a completely monodisperse sample up to 1.0 for a polydisperse suspension. The zeta potential was determined by laser Doppler electrophoresis in a Zetasizer Nano Z (Malvern Instruments Inc, Malvern, UK).

### Cell line and culture

The canine CLBL-1 B-cell lymphoma cell line (provided by Dr. Barbara Rütgen, Department of Pathobiology, University of Veterinary Medicine, Vienna, Austria) (31, 32) was cultured in Roswell Park Memorial Institute-1640 (RPMI 1640) medium (Gibco, Thermo Fisher Scientifics, Waltham, MA, USA) supplemented with 10% heat inactivated fetal calf serum (FCS, Gibco) and penicillin 100 U/ml/streptomycin 0.1 mg/ml (Gibco) at 37°C in a humidified atmosphere of 5% CO_2_ (T75-tissue culture flasks, Greiner Bio-One, Kremsmünster, Austria).

### Immunoblotting

Cells were harvested, washed twice with PBS and lysed with RIPA lysis Buffer (25 mM TrisHCL pH 7.6, 150 mM NaCl, 1% NP-40, 1% sodiumdeoxycholate, 0.1% SDS) supplemented with protease inhibitor cocktail (Roche, Basel, Switzerland). An increasing amount of total protein cell extract was loaded onto 15% SDS—polyacrylamide gel electrophoresis (SDS-PAGE) and transferred to nitrocellulose membranes. The membranes were blocked in 5% non-fat milk in PBS containing 0.2% Tween-20. After blocking, the membranes were incubated with primary antibodies. To evaluate folate expression, membranes were incubated with folate receptor alpha antibody (1:500 dilution, 0.5 mg/ml, Invitrogen, Thermo Fisher scientific, Carlsbad, CA, USA) or anti-α-tubulin antibody (monoclonal, mouse, 1:1,250 dilution, Sigma-Aldrich). To assess acetylation of H3 histone, membranes were incubated with anti-acetylhistone H3 (Lys9, Lys14) antibody (polyclonal, rabbit, 1:2,500 dilution, Thermo Fisher Scientific, Rockford, IL, USA) or anti-histone H3 (polyclonal, rabbit, 1:1,000 dilution, Thermo Fisher Scientific). Membranes were then incubated with secondary antibody: Peroxidase-AffiniPure anti-rabbit IgG antibody (polyclonal, goat, 1:10,000 dilution, Jackson ImmunoResearch, PA, USA) or anti-mouse IgG HRP antibody (polyclonal, sheep, 1:7,500 dilution, Jackson ImmunoResearch) to assess folate expression and Peroxidase-AffiniPure anti-rabbit IgG antibody (polyclonal, goat, 1:10,000 dilution, Jackson ImmunoResearch, PA, USA), to evaluate H3 acetylation. Proteins were detected using Luminata Forte Western HRP (Merck Millipore, Billerica, MA, USA) and acquired using the ChemiDoc XRS+ imaging system (Bio-Rad, Hercules, CA, USA).

### Cytotoxic assay

To determine the effect of Pan loaded in non-targeted and FA-targeted liposomal formulations on CLBL-1 cell proliferation, a cell viability assay was performed using the Alamar blue cell viability (Invitrogen). Briefly, 6 × 10^5^ of cells were seeded in 96-well plates in 200 μl of culture medium and subjected to increasing doses (0.4–2,000 nM) of each PEG-Pan-Lip and FA-PEG-Pan-Lip formulations. Free Pan was used as a control. After 24 h treatment, cell viability was determined using Alamar Blue reagent, according to the manufacturer's instructions. Absorbance at 570 nm and 600 nm was measured using the iMark microplate Reader (Bio-Rad). Cell viability was calculated using the formula provided by the manufacturer. Two replicate wells were used to determine each data point and two independent experiments were carried out in different days. Best-fit EC_50_ values of each formulation were calculated using GraphPad Prism software (version 9.2.0, San Diego, CA, USA) using response vs. log (inhibitor) function with variable slope.

### Evaluation of apoptotic cell death

The percentage of apoptotic cells after treatment with each liposomal formulation was determined by flow cytometry using the Guava Nexin Assay. Cells were seeded and treated with increasing concentrations (1–20 nM) of liposome formulations loaded with Pan for 24 h. After treatment, cells were recovered, centrifuged at 500 g for 5 min and resuspended in PBS containing 2% FBS. Then, an equal volume of Guava Nexin reagent was added to 50 μl of the cell suspension and incubated for 20 min, at room temperature, protected from light. Guava easyCyte 5HT flow cytometer using the Nexin software module was used for sample acquisition and analysis.

Caspase-3 and 7 activities were measured using Caspase Glo 3/7 Assay (Promega, Madison, WI, USA). For this purpose, CLBL-1 cells were seeded and treated with 1–20 nM of each liposomal formulation loaded with Pan for 24 h. After treatment, 100 μl of each cell suspension was transferred into a white 96-well plate and 75 μl of caspase-Glo 3/7 reagent was added. The mixture was mixed by orbital shaking for 30 s and then incubated at room temperature for 30 min. Incubation allowed complete cell lysis, stabilization of cleavage of the proluminescent substrate mediated by caspases and an increase in the luminescent signal. Luminescence was measured using the GloMax-Multi+ Detection System (Promega).

### Cellular uptake by immunofluorescence and flow cytometry

To perform the qualitative analysis, 1.5 × 10^5^ of CLBL-1 cells were plated on ibidi μ-Slide 8 Well Glass Bottom (Ibidi, Fitchburg, WI, USA) and incubated for 24 h at 37°C in a humidified atmosphere of 5% CO_2_. Then, the rhodamine-labeled PEG-Lip and FA-PEG-Lip was added to the cells and incubated at 37°C for 3 and 6 h, respectively. After incubation, cells were washed twice with PBS, fixed with PFA 4% for 15 min at room temperature and washed twice. After washing, DAPI Vectashield (Vector Labs, Burlingame, CA, USA) was added to the cells. Image acquisition was performed on a confocal point-scanning Zeiss LSM 880 microscope (Carl Zeiss, Germany) equipped with a Plan-Apochromat DIC X63 oil objective (1.40 numerical aperture). Diode 405-30 laser was used to excite DAPI, and DPSS 561-20 laser to excite Rhodamine. In the Airyscan acquisition mode, × 1.80 zoom images were recorded at 1,024 × 1,024 resolution. ZEN software was used for image acquisition and Fiji software was used for image processing.

To determine the quantitative cellular uptake of PEG-Lip and FA-PEG-Lip formulations, flow cytometry was performed. Briefly, 1 × 10^6^ of CLBL-1 cells were incubated with 5 μmol/ml or 7.5 μmol/ml of the rhodamine-labeled PEG-Lip and FA-PEG-Lip in a complete medium without phenol red for 90 min, 3 h and 6 h at 37°C. The cells were centrifuged and washed twice with PBS to remove unbound liposomes. Data were collected and analyzed using the Attune NxT flow cytometer (Thermo Scientific).

### Animals

All animal-handling procedures were performed in accordance to EU recommendations for good practices and animal welfare and were approved by the Animal Care and Ethical Committee of the Faculty of Veterinary Medicine (Protocol_0050132016). All methods were performed in accordance with the relevant guidelines and regulations. Female 6–8-week-old SOPF/SHO SCID mice or CD1 mice were purchased from Charles River. Immunodeficient mice were maintained in microisolation cages under pathogen-free conditions. CD1 mice were maintained under standard conditions. Room conditions included a room temperature of 24–26°C and a cycle of 12 h light and 12 h of darkness. Food and water were sterilized and provided *ad libitum*.

### Preparation of ^111^In-liposomes

The diethylenetriaminepentaacetic acid(DTPA)-containing liposomes were labeled with Indium-111 (^111^In) upon incubation of the respective liposome with ^111^In-8-hydroxyquinoline (oxine) following a modified procedure of the literature (33). The radiolabeling of oxine involved, firstly, the preparation of an ethanolic solution of oxine (250 μL, 13.8 mM), which was diluted with 0.4 M acetate buffer pH 5.5 (1,000 μL). The resulting oxine solution was added to indium (^111^In) chloride (290 μL, 370 MBq/mL, Mallinckrodt/Curium, The Netherlands) and then incubated at room temperature for 15 min. The lipophilic components were extracted with dichloromethane (3 × ) and then evaporated to dryness under a gentle stream of nitrogen. The radiochemical yield was generally >95 % ^111^In-oxine as determined by instant thin-layer chromatography using glass microfiber chromatography paper impregnated with silica gel (iTLC-SG, Agilent Technologies) and ethanol as eluent. The obtained dry residue containing ^111^In-oxine was firstly dissolved in ethanol (30 μL) and phosphate-buffered saline (PBS) pH 7.4 (80 μL) was added. The resulting mixture was incubated with the DTPA-containing liposomes (300–1.5 mL) for 45 min at 37°C. The ^111^In-liposomes were purified by ultrafiltration using a centrifugal concentrator—Amicon Ultra-0.5 Centrifugal Filter Unit (100 kDa MWCO, 0.5 mL sample volume, Merck) following the manufacturer's instructions. The labeling efficiency, which varied between 51% and 80% depending on the type of liposome, was determined by dividing the radioactivity from the concentrate that corresponds to ^111^In-liposome by the total amount loaded onto the Amicon filter.

### Biodistribution studies in CD1 mice

To evaluate the biodistribution in healthy mice, targeted and non-targeted ^111^In-labeled liposomes diluted in PBS (100 μL) were injected intravenously via the tail vein into CD1 mice. At 1 h, 3 h and 24 h post-injection (p.i.), mice were sacrificed by cervical dislocation. The injected radioactivity dose and the radioactivity in the sacrificed animal were measured using a dose calibrator (Carpintec CRC-15W, Ramsey, USA). The difference between the radioactivity in the injected dose and the sacrificed animals was accepted to be due to excretion. After sacrificed, tissue samples were collected, rinsed with PBS, weighed, and counted in a gamma counter (Hidex AMG, Hidex, Turku, Finland). Results are expressed as the mean percentage of the injected dose (ID) per gram of tissue (%ID/g tissue) (mean ± SD) (*n* = 3 per liposomal formulation).

### Tumor induction, biodistribution, and tumor targeting in SCID mice

For tumor induction, 1 × 10^6^ CLBL-1 cells diluted in PBS and matrigel (1:1) (Corning, NY, USA, Cat) were subcutaneously injected into the dorsal interscapular region of SCID mice as previously described (34). Tumor volume was calculated using the formula (width)^2^ x length. When the tumor reached a minimum volume of 150 mm^3^, the mice were randomized and divided into two distinct groups (targeted and non-targeted ^111^In-labeled liposomal formulations). Subsequently, the radiolabeled liposomes were intravenous injected into SCID mice. After sacrifice, the tissues were dissected and counted in a gamma counter, at different time points (24 h and 48 h p.i.). Tumor and tissue uptake were expressed as percentage of the injected dose per gram of tissue (%ID/g).

### Histopathological analysis

Tumors were fixed in 10% buffered formalin and embedded in paraffin utilizing a Leica tissue processor. Sections were cut from paraffin blocks and stained with hematoxylin and eosin (H&E). sections were mounted onto superfrost ultra plus slides (Menzel-Glaser, Braunschweig, DE) for immunohistochemistry.

### Immunohistochemistry analysis

A representative area of each tumor was selected and tissue sections of 3 μm thickness were mounted on glass slides (Superfrost glass slides, Thermo Scientific, Braunschweig, Germany), deparaffinized with xylene and hydrated in a graded ethanol series of distilled water. The Novolink Polymer Detection System (Noocastra, Leica Biosystems, Newcastle, UK) was used according to the manufacturer's instructions. The antigen retrieval treatment was achieved by microwave treatment (5 min at 900 watts plus 15 min at 650 watts) in Tris-EDTA buffer (pH 9.0). To block endogenous peroxidase and to prevent unspecific labeling, the system's Peroxidase Block Solution and Protein Block Solution were used sequentially. Sections were incubated 30 min at room temperature with polyclonal rabbit anti-human CD20 (Thermo Fisher Scientific), diluted 1:200 and rabbit polyclonal anti-human CD3 (Dako, Glostrup, Denmark), diluted 1:400. Labeling was developed by incubating the slides with system's chromogen, diaminobenzidine (DAB), and hydrogen peroxide as substrate. Nuclear background staining was performed with Gill's hematoxylin (30 s). Labeling without the primary antibody was used as negative control, while dog lymph node sections were used as positive control.

### Statistical analysis

Results are expressed as mean ± standard deviation (SD) or mean ± standard error mean (SEM). Statistical analysis was performed using one-way ANOVA and two-tailed Student's *t*-test using GraphPad Prism^®^ 9 (GraphPad Software, CA, USA). *P* < 0.05 was considered statistically significant.

## Results

### Physicochemical properties of panobinostat loaded liposomes are suitable for drug delivery

Owing to the potent anticancer activity of Pan on canine B-cell lymphoma demonstrated previously by our group (21, 34), this HDACi was selected for the study described herein. Although Pan exhibited promising cytotoxicity and antitumor properties *in vitro* and *in vivo*, it has been associated with significant dose-limiting toxicities, which might lead to some limitations for its clinical translation and safety as a single/adjuvant agent. Therefore, there is an urgent need to mitigate the high toxicity of Pan, as well as other HDACis. One of the best strategies to overcome this issue is to explore drug delivery systems. Within this context, in the present study we aimed to develop a Pan loaded PEGylated liposome drug delivery system with improved therapeutic outcomes. For this purpose, unloaded and loaded folate-targeted (FA-PEG-Lip and FA-PEG-Pan-Lip) and non-targeted liposomal formulations (PEG-Lip and PEG-Pan-Lip) were prepared, characterized and their biological activity tested. All liposomal formulations were prepared using the dehydration-rehydration method followed by an extrusion step to reduce and homogenize the mean size of the liposomes. The physicochemical properties of the liposomes, namely particle size, zeta potential, and incorporation parameters, such as encapsulation efficiency and loading capacity, are listed in Table 1. All liposome formulations presented a mean size of 130 nm and a PI < 0.2, demonstrating the high homogeneity of the so developed liposomes. The Zeta potential revealed a neutral surface charge under all the conditions being in accordance with the presence of DSPE-PEG at liposomal surface. Regarding Pan incorporation parameters, liposomes loaded with folate presented a higher encapsulation efficiency (94%), with a final loading capacity of 33 μg/μmoL than non-targeted liposomes (21 μg/μmoL). Nevertheless, all formulations were selected to study their cytotoxic and targeting properties against canine B-cell lymphoma.

**Table 1:** Characterization of target and non-targeted liposomes unloaded and loaded with Pan.

**Lipid composition (molar ratio)**	**(Pan/Lip)i (μg/μmol)**	**(PAN/Lip)f (μg/μmol)**	**E.E. (%)**	**Mean size (nm) (P.I.)**	**Zeta pot (mV)**
PEG-Pan-Lip; DPPC:Chol:DSPE-PEG (1.85:1.0.15)	39 ± 1	21 ± 1	56 ± 2	130 (< 0.050)	−2 ± 1
FA-PEG-Pan-Lip; DPPC:Chol:DSPE-PEG: DSPE-PEG-FA (1.85:1.0.12:0.03)	35 ± 1	33 ± 1	94 ± 2	130 (< 0.050)	−2 ± 1
PEG- Lip; DPPC:Chol:DSPE-PEG (1.85:1.0.15)	na	na	na	130 (< 0.070)	−3 ± 1
FA-PEG-Lip; DPPC:Chol:DSPE-PEG: DSPE-PEG-FA (1.85:1.0.12:0.03)	na	na	na	130 (< 0.070)	−3 ± 1

FA, Folate; (PAN/Lip)i, initial Pan to lipid ratio; (PAN/Lip)f, final Pan to lipid ratio; E.E., encapsulation efficiency; na, not applicable; P.I., polydispersity index.

### Folate receptor is expressed in canine lymphoma cells

**Figure 1:**
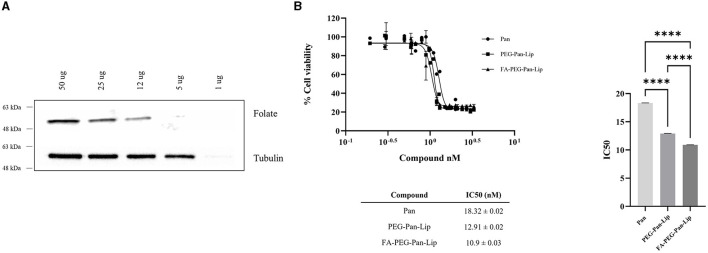
Evaluation of folate receptor expression and cytotoxic activity of folate-targeted and nontargeted liposomes loaded with panobinostat was evaluated in CLBL-1 cells. **(A)** Folate receptor expression was evaluated in total cell extracts from CLBL-1 cells using an anti-folate receptor antibody. Loading was controlled with an anti-tubulin antibody. Representative blots are shown. **(B)** Cells were treated with increasing concentrations of liposomes. After 24 h treatment, cell viability was measured using Alamar Blue reagent. Two replicate wells were utilized to determine each data point and three independent experiments were carried out in different days. Best-fit IC50 values of each formulation were calculated using the log (inhibitor) vs response (variable slope) function. Statistical significance was determined with one-way ANOVA followed by a Tukey's test. Values of *p* < 0.05 were considered significant. ^****^p < 0.0001.

The folate receptor is overexpressed in cancer cells, making it a suitable molecular target for specific drug delivery (9). Therefore, to assess the feasibility of using the folate receptor as a target for canine B-cell lymphoma, we evaluated its expression by western blot analysis in the well-characterized CLBL-1 canine lymphoma cell line (31, 32). Immunoblotting analysis (Figure 1A) confirmed folate receptor expression in the canine DLBCL cell line and demonstrated an increasing presence of the receptor, in agreement with the increasing amount of cellular extract. These results confirmed the presence of the folate receptor in CLBL-1, allowing us to explore it as a promising target and to evaluate the cytotoxic and targeting properties of the different liposome formulations prepared.

### Panobinostat-loaded liposomes present cytotoxicity in canine B-cell lymphoma

To evaluate the potential cytotoxic activity of the different liposome formulations in canine B-cell lymphoma, we conducted a cell viability assay in the CLBL-1 cell line. Cell viability of lymphoma cells subjected to a 24 h treatment with non-targeted and folate-targeted liposomes loaded with Pan was evaluated using Alamar Blue reagent, as described in the Materials and Methods section. PEG-Lip, FA-PEG-Lip and free Pan were used as controls. As shown in Figure 1B, Pan liposome formulations (PEG-Pan-Lip and FA-PEG-Pan-Lip) exhibited a dose-dependent inhibitory effect on CLBL-1 cell proliferation. In contrast, no cytotoxicity was observed for the PEG-Lip and FA-PEG-Lip formulations (data not shown). The differences between the IC_50_ values for each liposomal formulation and Pan-free were statistically significant. Moreover, the cytotoxicity of Pan was potentiated after incorporation in liposomes, probably due to a higher internalization in tumor cells. Importantly, the obtained data have shown that the IC_50_ values were in the nM range and that the Pan folate-targeted liposomal formulation seems to exhibit a slightly higher cytotoxic effect than the non-target liposomal formulation and Pan-free (FA-PEG-Pan-Lip, IC_50_ = 10.9 ± 0.03 nM, PEG-Pan-Lip, IC_50_ = 12.91 ± 0.02 nM and Pan-free, IC_50_ = 18.32 ± 0.024 nM).

### Panobinostat-loaded liposomes induce H3 histone acetylation and apoptosis

**Figure 2:**
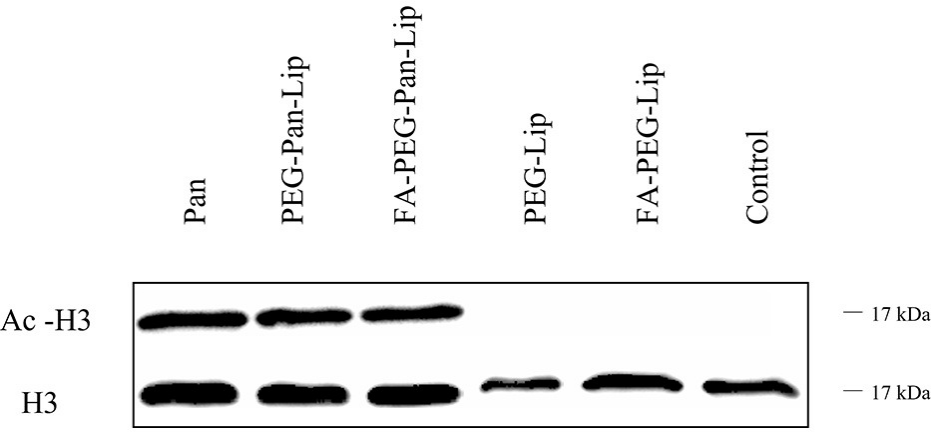
Assessment of H3 Histone Acetylation. H3 histone acetylation was assessed in total cell extracts from CLBL-1 cells after 24 h treatment with 20 uM of folate-targeted liposomes and nontargeted liposomes loaded with Panobinostat. Acetylation of H3 histones was evaluated by western blotting with an anti-acetyl-histone H3 polyclonal antibody. As loading control, H3 histone was assessed using anti-histone H3 polyclonal antibody. Representative blots are presented.

**Figure 3:**
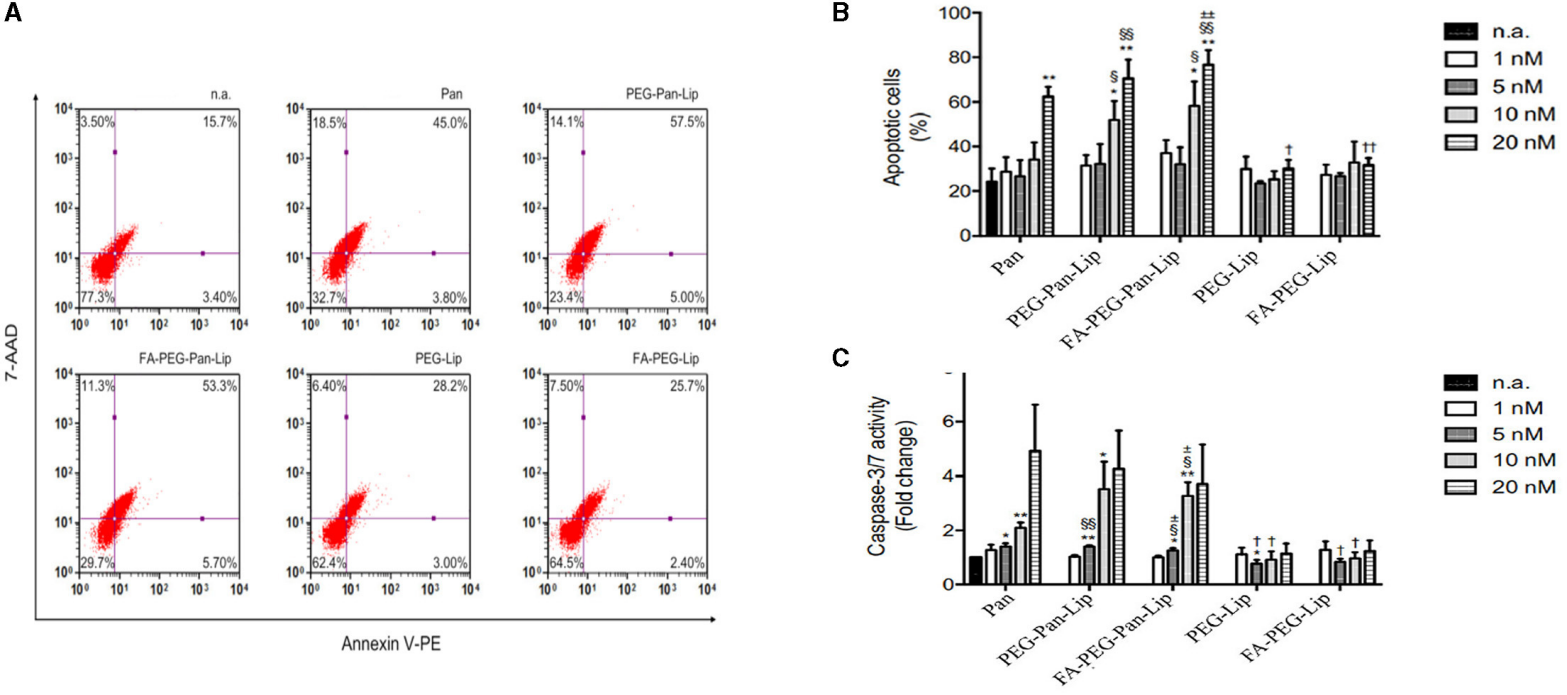
Evaluation of apoptotic cell death. **(A)** CLBL-1 cells were treated with 20 nM folate-targeted and nontargeted liposomes loaded with panobinostat for 24 h and representative flow cytometry plots using Annexin V/7-AAD staining are shown. **(B)** The percentage of apoptotic cells was determined in CLBL-1 cells subjected to a range of concentrations of folate-targeted and nontargeted liposomes loaded with panobinostat. After 24 h treatment, apoptotic cells were determined by flow cytometry using the Guava Nexin Assay. **(C)** Caspase 3/7 activity was evaluated in CLBL-1 cells subjected to increasing concentrations of folate-targeted and nontargeted liposomes loaded with panobinostat. After 24 h treatment, activity was determined using the Caspase-Glo 3/7 assay. Results are expressed as means ± SEM fold change to control cells. *p < 0.05 and **p < 0.01 from n.a.; ^†^*p* < 0.05 and ^††^*p* < 0.005 from Pan; ^§^*p* < 0.05 and ^§§^*p* < 0.01 from PEG-Lip; ^±^*p* < 0.05 and ^±±^*p* < 0.001 from FA-PEG-Lip. Statistical analysis was performed using Student's t-test. Values of *p* < 0.05 were considered significant. n.a., no addition.

Pan alters gene expression by inducing the acetylation of histones at an early stage, causing several effects on the cell cycle and resulting in cell death. Thus, to validate the mechanism of action of Pan-loaded liposomes in CLBL-1 cells, we evaluated the acetylation status of H3 histones by western blot. The acetylation status of cells treated with liposomes loaded with Pan were compared to unloaded liposome formulations. Immunoblotting analysis (Figure 2) demonstrated that CLBL-1 cells presented a hyperacetylation status, after 24 h of treatment with PEG-Pan-Lip, FA-PEG-Pan-Lip formulations, when compared with PEG-Lip and FA-PEG-Lip formulations and vehicle-/control-treated cells. Cell death occorred by apoptosis (Figures 3A, B). These results are in agreement with the cell viability and proliferation data upon Pan treatment, indicating that the cytotoxic activity of Pan in the CLBL-1 cell line is consistent with the induction of apoptosis. To confirm that apoptosis is a central mechanism of Pan-loaded liposome-induced cell death, the caspase 3/7 activity levels and the percentage of apoptotic cells after 24 h of treatment were determined. The results shown in Figure 3C indicate that caspase 3/7 activity was promoted in a dose-dependent manner by the PEG-Pan-Lip and FA-PEG-Pan-Lip formulations and the maximum caspase-3/7 activity was seen at 20 nM.

### Uptake of liposome formulations in CLBL-1 cells

**Figure 4:**
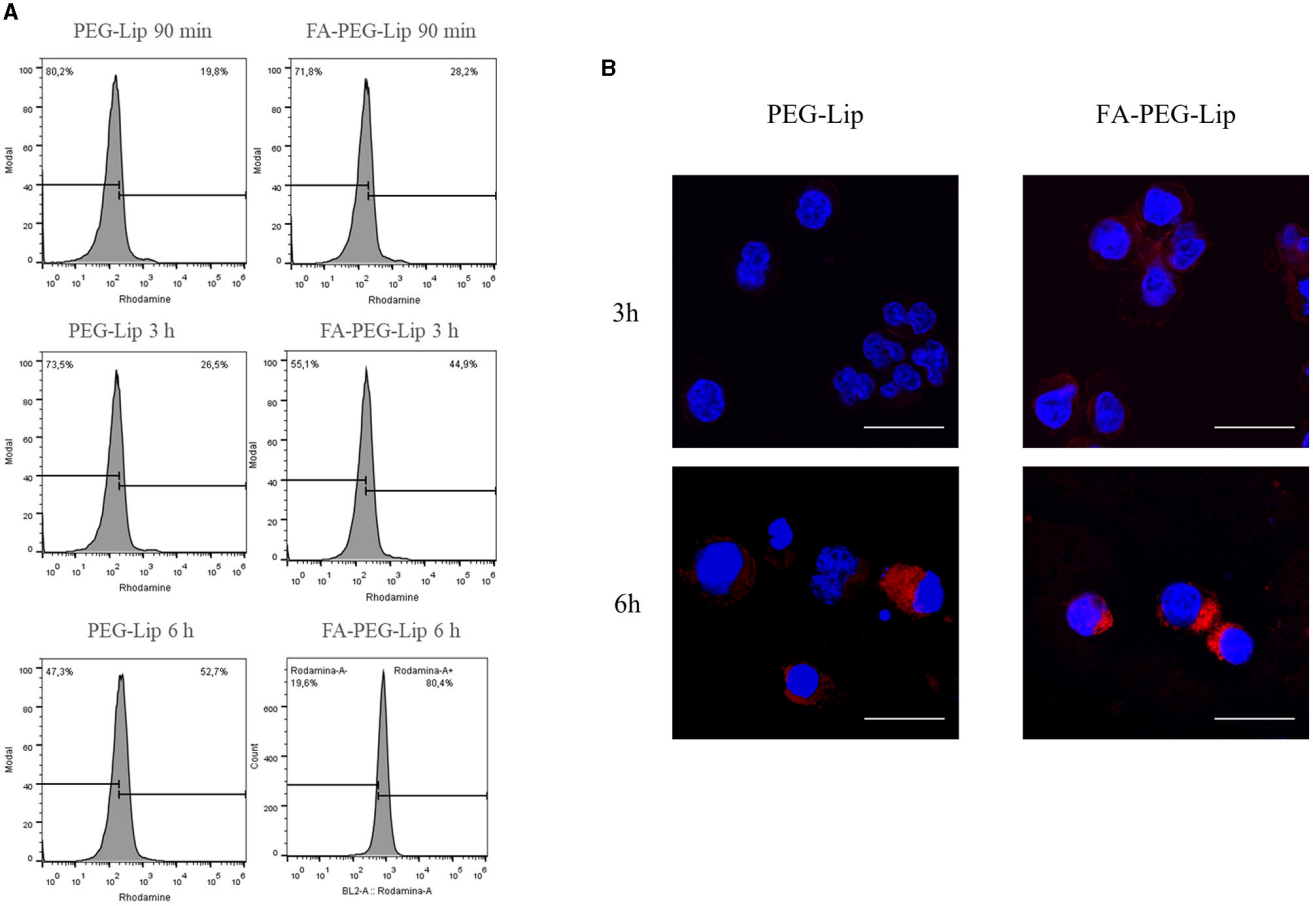
Evaluation of cellular uptake by flow cytometry and immunofluorescence. **(A)** To evaluate the quantitative cellular uptake of the liposomes, flow cytometry was performed. 1 × 10^6^ of CLBL-1 cells were incubated with 5 μmol/ml of PEG and PEG Folate labeled with phosphatidyl ethanolamine covalently linked to rhodamine for 90 min, 3 h and 6 h. Cellular uptake seems to be time dependent, achieving a high value after 6 h of incubation. Moreover, the percentage of uptake was higher in the formulation containing folate. **(B)** To determine the qualitative analysis, immunofluorescence was performed. 1 × 10^5^ of CLBL-1 cells were incubated for 3 h and 6 h with liposomes labeled with rhodamine. An accumulation of liposomes in the perinuclear area, confirmed the internalization of both formulations.

Intracellular uptake of rhodamine-labeled liposome formulation in CLBL-1 cells was evaluated by flow cytometry and immunofluorescence. For flow cytometry, labeled liposomes were incubated with CLBL-1 cells at different time points (90 min, 3 h and 6 h). The uptake of both formulations (FA-PEG-Pan-Lip and PEG-Pan-Lip) differed significantly, as shown in Figure 4A. The uptake was higher in the formulation containing folate at all time-points tested, demonstrating the importance of folate in facilitating cellular uptake. Additionally, the cellular uptake seemed to be time-dependent, since the amount of liposomes increased with time, reaching the highest value after 6 h incubation. Live/dead reagent was used to exclude dead cells, and background noise was evaluated in the control with the secondary antibody (data not shown). To better characterize the uptake efficiency of all liposomal formulations in CLBL-1 cells, we further evaluated the cellular internalization properties of the liposomes using confocal point-scanning microscopy after staining the nucleus with DAPI. As shown in Figure 4B, liposomes accumulated in the perinuclear area, confirming the internalization of both formulations. Moreover, FA-PEG-Pan-Lip significantly increased the fluorescent signal in the perinuclear region, in comparison with the PEG-Pan-Lip.

### Biodistribution studies in CD1 mice and xenograft mice model of canine B-cell lymphoma

To assess the *in vivo* stability, pharmacokinetic and tumor uptake profile of non-targeted and folate-targeted liposome formulations, we performed a biodistribution study in CD1 mice and in SCID xenograft mouse model of CLBL-1, respectively. For that purpose, FA-PEG-Pan-Lip and PEG-Pan-Lip formulations were radiolabeled with ^111^In, according to a previously reported procedure (29, 35). Both ^111^In-lipossomes were intravenously administrated to CD1 or to SCID mice and the biodistribution was evaluated at different time points. The biodistribution data of ^111^In-labeled PEG-Pan-Lip and FA-PEG-Pan-Lip, expressed as %ID/g of the main tissues and tumors, are presented in Tables 2, 3. Analysis of the data, in the CD1 mouse model, revealed that both ^111^In-liposomal preparations presented a similar tissue distribution profile with a moderate blood clearance (12.7 ± 3.8, 3.5 ± 0.6, 0.67 ± 0.08 %ID/g for ^111^In-PEG-Pan-Lip and 12.8 ± 0.7, 5.4 ± 0.7, 1.2 ± 0.6 %ID/g for ^111^In-FA-PEG-Pan-Lip, at 1 h, 24 h and 48 h p.i., respectively). Moderate hepatic uptake was found (1.1 ± 0.6, 5.9 ± 0.5/g of liver for ^111^In-PEG-Pan-Lip and 2.19 ± 0.05, 6.8 ± 1.4 %ID/g for ^111^In-FA-PEG-Pan-Lip, at 1 h, 24 h, respectively) that slightly decreased at 48 h indicating the hepatobiliar path as the main elimination route. However, the involvement of the urinary excretory pathway is also evident in the kidney uptake and in the whole-body radioactivity excretion rate. In fact, the untargeted formulation (^111^In-PEG-Pan-Lip) had a low kidney uptake (< 2.7 ± 0.3 %ID/g of kidney) associated to a rapid total excretion (59.7 ± 6.6, 68.3 ± 0.4, 82.3 ± 4.2 %ID, at 1 h, 24 h and 48 h p.i., respectively). The kidney uptake of the targeted formulation (^111^In-FA-PEG-Pan-Lip) increased over time (1.7 ± 0.9, 5.6 ± 0.3, 4.1 ± 2.7 %ID/g of kidney, at 1 h, 24 h and 48 h p.i., respectively) probably due to the presence of the folate moiety in the liposomes since the high expression of folate receptors in the renal proximal tubules is known. Consequently, the rate of total excretion is lower, ~30%, at 1 h p.i. The washout from major organs, except spleen was also rapid in both formulations. Liposomes were promptly eliminated from the heart, intestine, lungs, intestines and stomach. Radioactivity accumulation of radiolabeled liposomes was observed in the spleen (1.7 ± 0.4, 12.5 ± 4.2, 11.2 ± 3.9 %ID/g for ^111^In-PEG-Pan-Lip and 2.6 ± 1.1, 7.8 ± 1.5, 8.2 ± 1.6 %ID/g for ^111^In-FA-PEG-Pan-Lip, at 1 h, 24 h and 48 h p.i., respectively) reflecting the expected uptake from the mononuclear phagocyte system. Regarding the biodistribution and tumor uptake in the SCID xenograft mouse model of CLBL-1, the trend of the biodistribution profile is similar. Moderate blood clearance associated to hepatic and splenic uptake. Higher kidney uptake and lower rate of total excretion of ^111^In-FA-PEG-Pan-Lip than ^111^In-PEG-Pan-Lip. Moreover, and importantly, this preliminary biodistribution study demonstrated the ability of the targeted formulation (^111^In-FA-PEG-Pan-Lip) to accumulate in FR-expressing tumors. Indeed, the tumor uptake was 1.6-fold higher at 24h p.i (2.2 ± 0.9 %ID/g of tumor) than the ^111^In-PEG-Pan-Lip formulation (1.32 ± 0.2 %ID/g of tumor), as shown in Table 3.

**Table 2:** Biodistribution profiles of radiolabeled FA-PEG-Pan-Lip and PEG-Pan-Lip in healthy mice.

**Organ**	^**111**^ **In-PEG-Pan-Lip**			^**111**^ **In-FA-PEG-Pan-Lip**		
	**1 h**	**24 h**	**48 h**	**1 h**	**24 h**	**48 h**
Blood	12.7 ± 3.8	3.5 ± 0.6	0.67 ± 0.08	12.8 ± 0.7	5.4 ± 0.7	1.2 ± 0.6
Liver	1.1 ± 0.6	5.9 ± 0.5	4.5 ± 1.4	2.19 ± 0.05	6.8 ± 1.4	6.3 ± 1.6
Intestine	0.55 ± 0.06	0.98 ± 0.07	0.6 ± 0.2	1.0 ± 0.4	1.2 ± 0.2	1.1 ± 0.2
Spleen	1.7 ± 0.4	12.5 ± 4.2	11.2 ± 3.9	2.6 ± 1.1	7.8 ± 1.5	8.2 ± 1.6
Heart	0.7 ± 0.2	1.3 ± 0.3	0.7 ± 0.2	0.7 ± 0.4	1.9 ± 0.7	0.9 ± 0.2
Lung	1.0 ± 0.5	1.6 ± 0.6	0.6 ± 0.2	1.4 ± 0.6	2.2 ± 0.4	0.5 ± 0.3
Kidney	1.3 ± 0.6	2.7 ± 0.3	2.2 ± 0.0	1.7 ± 0.9	5.6 ± 0.3	4.1 ± 2.7
Muscle	0.4 ± 0.1	1.3 ± 0.9	0.26 ± 0.02	1.5 ± 0.1	0.7 ± 0.5	0.6 ± 0.1
Bone	0.6 ± 0.3	0.7 ± 0.3	0.6 ± 0.1	0.8 ± 0.2	1.0 ± 0.2	0.63 ± 0.02
Stomach	1.7 ± 0.9	1.1 ± 0.2	0.7 ± 0.3	1.2 ± 0.8	1.3 ± 0.7	0.8 ± 0.4
Brain	0.13 ± 0.03	0.16 ± 0.07	0.07 ± 0.05	0.17 ± 0.01	0.13 ± 0.03	0.04 ± 0.01
Carcass (%ID)	18.0 ± 0.9	26.0 ± 2.3	13.4 ± 0.7	37.2 ± 5.6	33.5 ± 3.9	28.8 ± 3.9
Excretion (%ID)	59.7 ± 6.6	68.3 ± 0.4	82.3 ± 4.2	25.7 ± 3.9	54.3 ± 1.4	63.5 ± 0.4

Radiolabeled liposomes were intravenous injected into CD1 mice. After sacrifice, the tissues were dissected and counted in a gamma counter, at different time points (1 h, 24 h and 48 h p.i.). Results are expressed as the mean percentage of the injected dose (ID) per gram of tissue (%ID/g tissue) (mean ± SD) (n = 3 per liposomal formulation).

**Table 3:** Biodistribution of radiolabeled folate-targeted and non-targeted liposomes in xenograft mice.

**Organ**	^**111**^ **In-PEG-Pan-Lip**		^**111**^ **In-FA-PEG-Pan-Lip**	
	**24 h**	**48 h**	**24 h**	**48 h**
Blood	1.8 ± 0.5	1.0 ± 0.3	4.6 ± 0.7	2.4 ± 0.4
Liver	5.0 ± 0.6	5.8 ± 1.6	11.9 ± 2.1	8.5 ± 2.0
Intestine	0.8 ± 0.2	0.73 ± 0.08	1.7 ± 0.1	1.6 ± 0.2
Spleen	20.6 ± 2.4	25.8 ± 0.5	16.8 ± 4.1	12.4 ± 4.4
Heart	0.32 ± 0.07	0.40 ± 0.02	1.2 ± 0.3	0.9 ± 0.2
Lung	0.68 ± 0.07	0.7 ± 0.2	2.3 ± 0.7	1.1 ± 0.6
Kidney	2.5 ± 0.3	1.9 ± 0.5	4.6 ± 0.9	4.4 ± 1.6
Muscle	0.12 ± 0.06	0.3 ± 0.2	0.7 ± 0.4	0.6 ± 0.4
Bone	0.3 ± 0.2	0.29 ± 0.07	0.8 ± 0.1	0.6 ± 0.1
Stomach	0.5 ± 0.2	0.40 ± 0.07	1.5 ± 0.7	1.0 ± 0.2
Brain	0.05 ± 0.02	0.04 ± 0.01	0.2 ± 0.1	0.05 ± 0.02
Tumor	1.3 ± 0.2	2.08 ± 0.09	2.2 ± 0.9	2.5 ± 0.8
Carcass (%ID)	14.5 ± 1.3	12.1 ± 1.2	34.4 ± 4.9	31.8 ± 3.5
Excretion (%ID)	69.8 ± 0.2	73.0 ± 1.1	27.8 ± 6.2	39.0 ± 9.1

Radiolabeled liposomes were intravenous injected into CD1 mice. After sacrifice, the tissues were dissected and counted in a gamma counter, at different time points (1 h, 24 h and 48 h p.i.). Results are expressed as the mean percentage of the injected dose (ID) per gram of tissue (%ID/g tissue) (mean ± SD) (n = 3 per liposomal formulation).model of cNHL. Radiolabeled liposomes were intravenous injected into SCID mice. After sacrifice, the tissues were dissected and counted in a gamma counter, at different time points (24 h and 48 h p.i.). Results are expressed as the mean percentage of the injected dose (ID) per gram of tissue (%ID/g tissue) (mean ± SD) (n = 3 per liposomal formulation).

**Figure 5:**
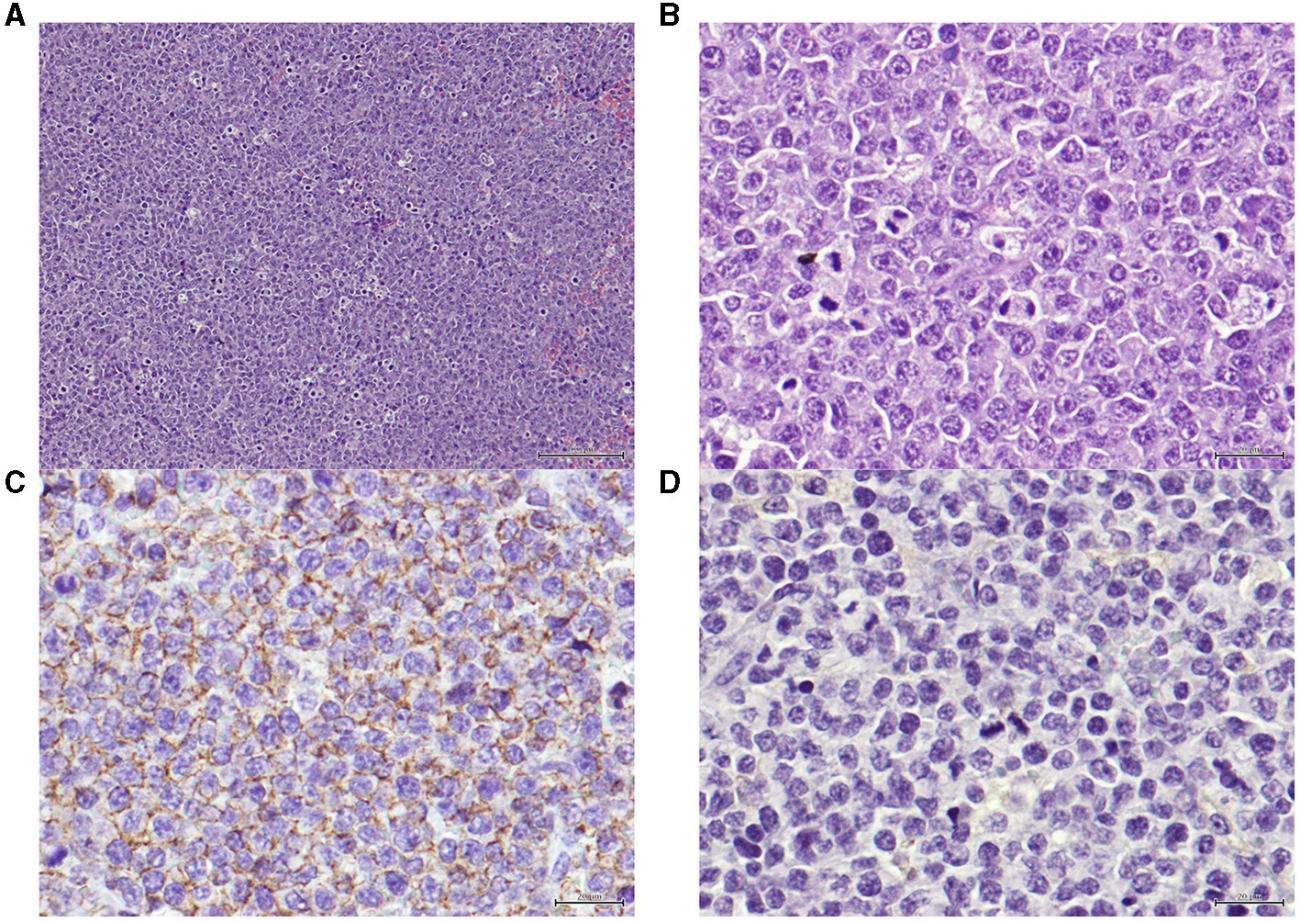
**(A)** Xenograft tumor section presenting a neoplasia, classified as high grade centroblastic diffuse malignant lymphoma. The neoplasia consists of monomorphic large cells with a high cell density and a starry-sky pattern. Hematoxylin and eosin (H&E) stained. Magnification = 100× , scale bar = 100 μm. **(B)** Xenograft tumor section presenting a lymphoma. The neoplastic is composed by monomorphic round cells, with several marginal and small nucleoli per cell and high mitotic index. Hematoxylin and eosin (H&E) stained. Magnification = 400×, scale bar = 20 μm. **(C)** Xenograft tumor section presenting the immunohistochemistry technique for B-cells, exhibiting positive staining on the cellular membrane level in virtually 100% of the tumor. Anti-CD20 antibody, Gill's hematoxylin. Magnification = 400×, scale bar = 20 μm. **(D)** Xenograft tumor section presenting the immunohistochemistry technique for T-cells, showing that the tumor cells are negative for this marker (anti-CD3, Gill's hematoxylin, 100x). Anti-CD3 antibody, Gill's hematoxylin. Magnification = 400×, scale bar = 20 μm.

Moreover, it is important to mention that histological and immunohistochemical analysis demonstrated that xenograft tumors maintained histological features and expression of B-cell markers were positive and expression of T-cell markers were negative, reflecting those of the original CLBL-1 cell line xenografts (Figure 5).

## Discussion

Lymphoma and NHL, in particular, are responsible for millions of deaths worldwide, representing a disturbing global health problem. The similarities between human NHL and canine lymphoma make NHL a transversal disease for both species, opening new opportunities to explore the advantages of translational research. In the present study, we explored a novel liposome-based drug delivery system to enhance the therapeutic benefits of Pan, a validated anticancer drug in comparative medicine (34). Pan is a cytotoxic compound belonging to the HDACi class that has shown great promise in relapsed DLBCL patients, inducing long-lasting durable responses in a phase 2 clinical study (36). Recently, we demonstrated its anticancer activity against canine B-cell lymphoma (34). However, this study revealed some *in vivo* toxic effects that can limit its clinical progression as a treatment option for canine lymphoma. The primary goal of anticancer therapy in veterinary medicine is to provide the best quality of life for as long as possible, as such dogs are not good candidates for aggressive regimens independent of their curability potential. This is also a concern in human medicine, considering that the potential of these molecules as anticancer therapeutics has been hampered by toxicity- and specificity-related issues. Although a multitude of drugs acting via HDAC inhibition are currently in clinical trials or in the market, only HDACis, such as vorinostat, romidepsin, and belinostat have been approved for some T-cell lymphomas and Pan for multiple myeloma. In addition to its non-specificity, HDACis, including valproic acid, trichostatin A, sodium butyrate, and vorinostat, are associated with clinical toxic effects, such as thrombocytopenia, nausea, and fatigue. Furthermore, vorinostat and romidepsin, two FDA-approved HDACis, are reported to have no partial or complete response in solid tumors and are linked to severe cardiac toxicity. Thus, in the present study, we hypothesized that the encapsulation of Pan into liposome nanocarriers could improve their therapeutic index and further reduce associated systemic toxicity effects, extending their use in both human and veterinary clinical settings.

Due to their biological and technological advantages, liposomes have been considered in the past few years as promising drug delivery systems for cancer applications. Remarkable advances have been made and multiple biomedical applications of liposomes have been tested in clinical trials or have already been approved (37, 38). The first liposomal formulation used in human medicine was Doxil, a doxorubicin liposomal formulation, approved for the treatment of ovarian cancer, multiple myeloma, and HIV-associated Kaposi's sarcoma (39). Over the years, other formulations have been approved for cancer therapy, such as Myocet, Marqibo and Vyxeos (40). Many studies in veterinary medicine have reported the use of drugs encapsulated in liposomes. Doxorubicin liposomes have been tested in canine models to evaluate their pharmacokinetics, biodistribution, and safety profiles (41–43). These studies confirmed that Doxil did not induce cardiotoxicity or myelosuppression in dogs, one of the most important side effects of free doxorubicin, making it a viable therapeutic option (41, 42). Another pilot study conducted by Hauck et al. reported results from a phase I clinical trial in dogs with spontaneous tumors, namely sarcomas and carcinomas, using low-temperature doxorubicin-loaded liposomes. Of the 21 patients enrolled in the study, 12 presented with stable disease and six had a partial response to the treatment. This study showed favorable clinical responses, validating a novel approach of a liposome-based delivery system for veterinary use (44).

Within this context, in the present study we aimed to develop Pan-loaded folate-targeted PEGylated liposomes with improved therapeutic outcomes for the treatment of canine B-cell lymphoma. For this purpose, non-targeted and folate-targeted PEGylated liposomal formulations were prepared and their cytotoxic and targeting properties against canine diffuse large B-cell lymphoma were thoroughly investigated. While non-targeted liposomes rely on enhanced permeability and retention to deliver the therapeutic agent to the tumor site, targeted liposomes are functionalized with surface ligands to improve selective tumor targeting and facilitate intracellular uptake. Due to its overexpression in a wide range of tumors, folate receptor targeting has shown great potential in mediating the tumor uptake of a variety of drugs (45). Several studies by Gabizon et al. demonstrated significant differences between non-targeted and folate-targeted liposomes in folate receptor-overexpressing tumors, including lymphoma (46, 47). This study compared the *in vivo* distribution of folate-targeted and non-targeted liposomes and found that folate-targeted liposomes were more effective than non-targeted liposomes in a lymphoma tumor model (47). The conclusions of this study were further reinforced by Shmeeda et al., who demonstrated intracellular uptake of folate-targeted liposomes in lymphoma cells (48). In another study, Gabizon et al. proved that folate-targeted liposomes loaded with doxorubicin were more effective than the non-targeted liposomes in a lymphoma model (46). More recently, Qiu et al. demonstrated the application of this drug delivery system in NHL by using vincristine-loaded lipid-polymer hybrid liposomes (VCR-loaded LPNs). This study reported a targeted effect in the delivery of FA-VCR-loaded LPNs toward B-cell lymphoma cells, with an outstanding therapeutic effect in the treatment of lymphoma, reducing systemic toxicity (49). Considering the high efficacy of drug-loaded folate-targeted liposomes in human lymphoma, we evaluated the folate receptor expression in canine diffuse large B-cell lymphoma and confirmed its overexpression in the CLBL-1 canine lymphoma B-cell line.

Pan liposomes were prepared using an active loading method and both formulations exhibited high incorporation parameters, particularly the one containing FA (PEG-Pan-Lip EE = 56± 2 % and FA-PEG-Pan-Lip EE = 94 ± 2 %). The methodology used in the present work, active loading, means that Pan was incorporated in pre-formed unloaded liposomes (10) in opposition to a passive loading where the compound is incorporated during liposome preparation (50). The active loading method presents several advantages over the passive methods namely stability and higher incorporation parameters as widely demonstrated in literature (51, 52). The pH or salt gradient differences between intra and extraliposomal membrane are the most known underlying mechanisms dictating the active drug loading. Moreover, active loading is based on the fact that uncharged drugs will cross the liposomal membrane and become protonated and entrapped inside the aqueous compartment of liposomes thus contributing to achieve high loadings and high stable liposomal formulations (53).

Liposomal formulations were further investigated *in vitro* to assess the suitability of their drug delivery properties. Firstly, cytotoxicity assays were performed to assess the effect of the liposomal formulations on the viability of canine diffuse large B-cell lymphoma cells. The data demonstrated that encapsulated Pan in both formulations (FA-PEG-Pan-Lip and PEG-Pan-Lip) maintained its cytotoxicity in CLBL-1 cells, when compared with Pan-free. The IC_50_ values determined for liposomal formulations were lower in comparison with Pan-free data, indicating that cytotoxic properties of the compound were not only preserved after incorporation in liposomes but potentiated. In addition, our data demonstrated that the cytotoxicity activity of the FA-PEG-Pan-Lip formulation was slightly higher than PEG-Pan-Lip formulation (FA-PEG-Pan-Lip IC_50_ = 10.9 ± 0.03 nM vs. PEG-Pan-Lip IC_50_ = 12.91 ± 0.02 nM). These data are in accordance with those reported in the literature, where folate is expected to enhance tumor uptake (45). Moreover, our data demonstrated that FA-PEG-Pan-Lip and PEG-Pan-Lip formulations were able to induce histone H3 acetylation in the CLBL-1 canine lymphoma cell line, the key molecular mechanism of HDACis.

HDAC inhibitors can induce multiple antitumor pathways. One of the main mechanisms of transformed cell death is the activation of apoptosis via intrinsic and extrinsic pathways (54). Activation of caspase-3 and 7 is an essential step during apoptosis and is used as a reliable marker for cells undergoing apoptosis (55). All liposomal formulations loaded with Pan at 20 nM demonstrated a high percentage of apoptotic cells and high levels of caspase-3 and 7 activation, similar to the data for the Pan-free formulation. Finally, to assess the *in vivo* stability and pharmacokinetic profile of each liposomal formulation, we performed biodistribution studies in CD1 mice and in a xenograft SCID mouse model of canine B-cell lymphoma. Biodistribution data demonstrated that FA-PEG-Pan-Lip formulation remained in circulation for a longer time, suggesting extended drug retention. In FA-PEG-Pan-Lip and PEG-Pan-Lip formulations, fast clearance from the major organs was observed, which is crucial to prevent systemic toxicity. However, a high accumulation of liposomal formulations was noted in the liver and spleen. Liposomes have specific clearance mechanisms from the bloodstream. The main mechanism is via recognition and uptake by macrophages of the reticuloendothelial system; consequently, the two main organs that present a major capacity for liposomal accumulation are the liver and the spleen (56). Thus, accumulation in the spleen and liver could be related to the elimination of liposomes. Importantly, the biodistribution data in the xenograft SCID mouse model of CLBL-1 have shown that the tumor uptake was higher with the targeted formulation (^111^In-FA-PEG-Pan-Lip) with a percentage of 2.2% and 2.5% ID/g of tumor at 24 h and 48 h, respectively. To the best of our knowledge, this is the first study to report the use of a liposome-based drug delivery system loaded with Pan in the treatment of canine B-cell lymphoma. Overall, these results validated that folate-targeted liposomes encapsulated with Pan can be a promising drug delivery system to be explored for a more effective and safer cancer treatment modality. Although this target-liposome-based drug delivery system showed strong cytotoxicity in canine lymphoma cells, additional preclinical studies in canine B-cell lymphoma xenograft murine models are needed to evaluate its *in vivo* efficacy and safety, to then allow its further progression to clinical studies in canine patients. In conclusion, this study contributes to the development of Pan nanocarriers for the treatment of canine B-cell lymphoma as a predictive preclinical surrogate for human NHL, mutually benefiting both species and opening up perspectives in comparative oncology.

## Data availability statement

The original contributions presented in the study are included in the article/supplementary material, further inquiries can be directed to the corresponding author.

## Ethics statement

The animal study was approved by Animal Care and Ethical Committee of the Faculty of Veterinary Medicine. The study was conducted in accordance with the local legislation and institutional requirements.

## Author contributions

AA and JD performed and analyzed the majority of the experiments and wrote the manuscript. SIA assisted with the cytotoxic assays, biodistribution studies, and reviewed the manuscript. AL and SN performed the flow cytometry experiments. JA and CR performed the apoptosis assays. MCav, VN, and MCas performed the immunofluorescence microscopy. CF, LG, and JDGC radiolabeled the liposomes and performed the biodistribution studies and reviewed the manuscript. JC performed the histopathological and immunohistochemistry analysis. MMG prepared and characterized liposomal formulations and reviewed the manuscript. LT supervised the work and reviewed the manuscript. FA-d-S was responsible for the research concept, experimental work supervision, and manuscript revision. All authors read and approved the final manuscript.

## References

[1] Sung H Ferlay J Siegel RL Laversanne M Soerjomataram I Jemal A. Global cancer statistics 2020: GLOBOCAN estimates of incidence and mortality worldwide for 36 cancers in 185 countries. CA Cancer J Clin. (2021) 71:209–49. 10.3322/caac.21660 33538338

[2] Gardner HL Fenger JM London CA. Dogs as a model for cancer. Annu Rev Anim Biosci. (2016) 4:199–222. 10.1146/annurev-animal-022114-110911 26566160 PMC6314649

[3] Schiffman JD Breen M. Comparative oncology: what dogs and other species can teach us about humans with cancer. Philos Trans R Soc Lond, B, Biol Sci. (2015) 370:20140231. 10.1098/rstb.2014.0231 26056372 PMC4581033

[4] Bray F Ferlay J Soerjomataram I Siegel RL Torre LA Jemal A. Global cancer statistics 2018: GLOBOCAN estimates of incidence and mortality worldwide for 36 cancers in 185 countries. CA Cancer J Clin. (2018) 68:394–424. 10.3322/caac.21492 30207593

[5] Bowzyk Al-Naeeb A Ajithkumar T Behan S Hodson DJ. Non-Hodgkin lymphoma. BMJ. (2018) 362:k3204. 10.1136/bmj.k3204 30135071

[6] Ito D Frantz AM Modiano JF. Canine lymphoma as a comparative model for human non-Hodgkin lymphoma: recent progress and applications. Vet Immunol and Immunopathol. (2014) 159:192–201. 10.1016/j.vetimm.2014.02.016 24642290 PMC4994713

[7] Ansell SM. Non-Hodgkin lymphoma: diagnosis and treatment. Mayo Clin Proc. (2015) 90:1152–63. 10.1016/j.mayocp.2015.04.025 26250731

[8] Aguiar SI Dias JNR André AS Silva ML Martins D Carrapiço B. Highly specific blood-brain barrier transmigrating single-domain antibodies selected by an *in vivo* phage display screening. Pharmaceutics. (2021) 13:1598. 10.3390/pharmaceutics13101598 34683891 PMC8540410

[9] Chaudhury A Das S. Folate receptor targeted liposomes encapsulating anti-cancer drugs. Curr Pharm Biotechnol. (2015) 16:333–43. 10.2174/1389201016666150118135107 25601598

[10] Gaspar MM Radomska A Gobbo OL Bakowsky U Radomski MW Ehrhardt C. Targeted delivery of transferrin-conjugated liposomes to an orthotopic model of lung cancer in nude rats. J Aerosol Med Pulm Drug Deliv. (2012) 25:310–8. 10.1089/jamp.2011.0928 22857016

[11] Torchilin VP. Passive and active drug targeting: drug delivery to tumors as an example. Handb Exp Pharmacol. (2010) 2010:3–53. 10.1007/978-3-642-00477-3_1 20217525

[12] Paoloni M Khanna C. Translation of new cancer treatments from pet dogs to humans. Nat Rev Cancer. (2008) 8:147–56. 10.1038/nrc2273 18202698

[13] Eckschlager T Plch J Stiborova M Hrabeta J. Histone deacetylase inhibitors as anticancer drugs. Int J Mol Sci. (2017) 18:1414. 10.3390/ijms18071414 28671573 PMC5535906

[14] Glozak MA Seto E. Histone deacetylases and cancer. Oncogene. (2007) 26:5420–32. 10.1038/sj.onc.1210610 17694083

[15] Chun P. Histone deacetylase inhibitors in hematological malignancies and solid tumors. Arch Pharm Res. (2015) 38:933–49. 10.1007/s12272-015-0571-1 25653088

[16] Sermer D Pasqualucci L Wendel H-G Melnick A Younes A. Emerging epigenetic-modulating therapies in lymphoma. Nat Rev Clin Oncol. (2019) 16:494–507. 10.1038/s41571-019-0190-8 30837715 PMC6650343

[17] Bertino EM Otterson GA. Romidepsin: a novel histone deacetylase inhibitor for cancer. Expert Opin Investig Drugs. (2011) 20:1151–8. 10.1517/13543784.2011.594437 21699444

[18] Lee H-Z Kwitkowski VE Del Valle PL Ricci MS Saber H Habtemariam BA. FDA approval: belinostat for the treatment of patients with relapsed or refractory peripheral T-cell lymphoma. Clin Cancer Res. (2015) 21:2666–70. 10.1158/1078-0432.CCR-14-3119 25802282

[19] Mann BS Johnson JR Cohen MH Justice R Pazdur R. FDA approval summary: vorinostat for treatment of advanced primary cutaneous T-cell lymphoma. Oncologist. (2007) 12:1247–52. 10.1634/theoncologist.12-10-1247 17962618

[20] Shah RR. Safety and tolerability of histone deacetylase (HDAC) inhibitors in oncology. Drug Saf. (2019) 42:235–45. 10.1007/s40264-018-0773-9 30649740

[21] Dias JNR Aguiar SI Pereira DM André AS Gano L Correia JDG. The histone deacetylase inhibitor panobinostat is a potent antitumor agent in canine diffuse large B-cell lymphoma. Oncotarget. (2018) 9:28586–98. 10.18632/oncotarget.25580 29983882 PMC6033347

[22] Co S Jo P Jm L Aj A Mm G C R. Current trends in cancer nanotheranostics: metallic, polymeric, and lipid-based systems. Pharmaceutics. (2019) 11:22. 10.3390/pharmaceutics11010022 30625999 PMC6359642

[23] De Souza C Ma Z Lindstrom AR Chatterji BP. Nanomaterials as potential transporters of HDAC inhibitors. Med Drug Discov. (2020) 6:100040. 10.1016/j.medidd.2020.10004017487363

[24] Ferreira M Ogren M Dias JNR Silva M Gil S Tavares L. Liposomes as antibiotic delivery systems: a promising nanotechnological strategy against antimicrobial resistance. Molecules. (2021) 26:2047. 10.3390/molecules26072047 33918529 PMC8038399

[25] Luiz H Oliveira Pinho J Gaspar MM. Advancing medicine with lipid-based nanosystems-the successful case of liposomes. Biomedicines. (2023) 11:435. 10.3390/biomedicines11020435 36830971 PMC9953160

[26] Bulbake U Doppalapudi S Kommineni N Khan W. Liposomal formulations in clinical use: an updated review. Pharmaceutics. (2017) 9:12. 10.3390/pharmaceutics9020012 28346375 PMC5489929

[27] Sercombe L Veerati T Moheimani F Wu SY Sood AK Hua S. Advances and challenges of liposome assisted drug delivery. Front Pharmacol. (2015) 6:286. 10.3389/fphar.2015.00286 26648870 PMC4664963

[28] Chaudhury A Das S Bunte RM Chiu GNC. Potent therapeutic activity of folate receptor-targeted liposomal carboplatin in the localized treatment of intraperitoneally grown human ovarian tumor xenograft. Int J Nanomedicine. (2012) 7:739–51. 10.2147/IJN.S26172 22359453 PMC3282613

[29] Gaspar MM Boerman OC Laverman P Corvo ML Storm G Cruz MEM. Enzymosomes with surface-exposed superoxide dismutase: *in vivo* behaviour and therapeutic activity in a model of adjuvant arthritis. J Control Rel. (2007) 117:186–95. 10.1016/j.jconrel.2006.10.018 17169460

[30] Pinho JO Amaral JD Castro RE Rodrigues CM Casini A Soveral G. Copper complex nanoformulations featuring highly promising therapeutic potential in murine melanoma models. Nanomedicine. (2019) 14:835–50. 10.2217/nnm-2018-0388 30875274

[31] Rütgen BC Hammer SE Gerner W Christian M de Arespacochaga AG Willmann M. Establishment and characterization of a novel canine B-cell line derived from a spontaneously occurring diffuse large cell lymphoma. Leuk Res. (2010) 34:932–8. 10.1016/j.leukres.2010.01.021 20153049

[32] Rütgen BC Willenbrock S Reimann-Berg N Walter I Fuchs-Baumgartinger A Wagner S. Authentication of primordial characteristics of the CLBL-1 cell line prove the integrity of a canine b-cell lymphoma in a murine *in vivo* model. PLoS ONE. (2012) 7:e40078. 10.1371/journal.pone.0040078 22761949 PMC3386195

[33] Yang F-Y Wang H-E Liu R-S Teng M-C Li J-J Lu M. Pharmacokinetic analysis of 111In-labeled liposomal doxorubicin in murine glioblastoma after blood-brain barrier disruption by focused ultrasound. PLoS ONE. (2012) 7:e45468. 10.1371/journal.pone.0045468 23029030 PMC3445513

[34] Dias JNR André AS Aguiar SI Ministro J Oliveira J Peleteiro MC. Establishment of a bioluminescent canine B-cell lymphoma xenograft model for monitoring tumor progression and treatment response in preclinical studies. PLoS ONE. (2018) 13:e0208147. 10.1371/journal.pone.0208147 30592723 PMC6310248

[35] Pinho JO Matias M Marques V Eleutério C Fernandes C Gano L. Preclinical validation of a new hybrid molecule loaded in liposomes for melanoma management. Biomed Pharmacother. (2023) 157:114021. 10.1016/j.biopha.2022.114021 36399831

[36] Assouline SE Nielsen TH Yu S Alcaide M Chong L MacDonald D Tosikyan A. Phase 2 study of panobinostat with or without rituximab in relapsed diffuse large B-cell lymphoma. Blood. (2016) 128:185–94. 10.1182/blood-2016-02-699520 27166360 PMC4972610

[37] Beltrán-Gracia E López-Camacho A Higuera-Ciapara I Velázquez-Fernández JB Vallejo-Cardona AA. Nanomedicine review: clinical developments in liposomal applications. Cancer Nanotechnol. (2019) 10:11. 10.1186/s12645-019-0055-y

[38] Bozzuto G Molinari A. Liposomes as nanomedical devices. Int J Nanomedicine. (2015) 10:975–99. 10.2147/IJN.S68861 25678787 PMC4324542

[39] Barenholz Y. Doxil^®^-the first FDA-approved nano-drug: lessons learned. J Control Release. (2012) 160:117–34. 10.1016/j.jconrel.2012.03.020 22484195

[40] Liu P Chen G Zhang J A. Review of liposomes as a drug delivery system: current status of approved products, regulatory environments, and future perspectives. Molecules. (2022) 27:1372. 10.3390/molecules27041372 35209162 PMC8879473

[41] Sorenmo K Samluk M Clifford C Baez J Barrett JS Poppenga R. Clinical and pharmacokinetic characteristics of intracavitary administration of pegylated liposomal encapsulated doxorubicin in dogs with splenic hemangiosarcoma. J Vet Intern Med. (2007) 21:1347–54. 18196746 10.1892/06-214.1

[42] Teske E Rutteman GR Kirpenstein J Hirschberger J. A randomized controlled study into the efficacy and toxicity of pegylated liposome encapsulated doxorubicin as an adjuvant therapy in dogs with splenic haemangiosarcoma. Vet Comp Oncol. (2011) 9:283–9. 10.1111/j.1476-5829.2011.00266.x 22077409

[43] Vail DM Kravis LD Cooley AJ Chun R MacEwen EG. Preclinical trial of doxorubicin entrapped in sterically stabilized liposomes in dogs with spontaneously arising malignant tumors. Cancer Chemother Pharmacol. (1997) 39:410–6. 10.1007/s002800050591 9054954

[44] Hauck ML. Phase I trial of doxorubicin-containing low temperature sensitive liposomes in spontaneous canine tumors. Clin Cancer Res. (2006) 12:4004–10. 10.1158/1078-0432.CCR-06-0226 16818699

[45] Kumar P Huo P Liu B. Formulation strategies for folate-targeted liposomes and their biomedical applications. Pharmaceutics. (2019) 11:381. 10.3390/pharmaceutics11080381 31382369 PMC6722551

[46] Gabizon A Tzemach D Gorin J Mak L Amitay Y Shmeeda H. Improved therapeutic activity of folate-targeted liposomal doxorubicin in folate receptor-expressing tumor models. Cancer Chemother Pharmacol. (2010) 66:43–52. 10.1007/s00280-009-1132-4 19779718

[47] Gabizon A Horowitz AT Goren D Tzemach D Shmeeda H Zalipsky S. *In vivo* fate of folate-targeted polyethylene-glycol liposomes in tumor-bearing mice. Clin Cancer Res. (2003) 9:6551–9. 14695160

[48] Shmeeda H. Intracellular uptake and intracavitary targeting of folate-conjugated liposomes in a mouse lymphoma model with up-regulated folate receptors. Mol Cancer Ther. (2006) 5:818–24. 10.1158/1535-7163.MCT-05-0543 16648551

[49] Qiu L Dong C Kan X. Lymphoma-targeted treatment using a folic acid-decorated vincristine-loaded drug delivery system. Drug Des Devel Ther. (2018) 12:863–72. 10.2147/DDDT.S152420 29713144 PMC5909786

[50] Gaspar MM Calado S Pereira J Ferronha H Correia I Castro H. Targeted delivery of paromomycin in murine infectious diseases through association to nano lipid systems. Nanomedicine. (2015) 11:1851–60. 10.1016/j.nano.2015.06.008 26169150

[51] Barenholz Y. (Chezy) “Doxil^®^–the first FDA-approved nano-drug: from an idea to a product. In: *Handbook of Harnessing Biomaterials in Nanomedicine*. Dubai: Jenny Stanford Publishing (2021)

[52] Nakhaei P Margiana R Bokov D Abdelbasset WK Kouhbanani M Varma RS. Liposomes: structure, biomedical applications, and stability parameters with emphasis on cholesterol. Front Bioeng Biotechnol. (2021) 9:705886. 10.3389/fbioe.2021.705886 34568298 PMC8459376

[53] Sur S Fries AC Kinzler W Zhou S Vogelstein B. Remote loading of preencapsulated drugs into stealth liposomes. Proc Natl Acad Sci U S A. (2014) 111:2283–8. 10.1073/pnas.1324135111 24474802 PMC3926059

[54] Xu WS Parmigiani RB Marks PA. Histone deacetylase inhibitors: molecular mechanisms of action. Oncogene. (2007) 26:5541–52. 10.1038/sj.onc.1210620 17694093

[55] Shim MK Yoon HY Lee S Jo MK Park J Kim J-H. Caspase-3/-7-specific metabolic precursor for bioorthogonal tracking of tumor apoptosis. Sci Rep. (2017) 7:16635. 10.1038/s41598-017-16653-2 29192289 PMC5709468

[56] Ait-Oudhia S Mager D Straubinger R. Application of pharmacokinetic and pharmacodynamic analysis to the development of liposomal formulations for oncology. Pharmaceutics. (2014) 6:137–74. 10.3390/pharmaceutics6010137 24647104 PMC3978529

